# Brain mitophagy in space and time

**DOI:** 10.1038/s44318-024-00275-2

**Published:** 2024-10-30

**Authors:** Vassiliki Nikoletopoulou

**Affiliations:** Department of Fundamental Neurosciences, University of Lausanne, Lausanne, Switzerland

**Keywords:** Autophagy & Cell Death, Neuroscience

## Abstract

A recent longitudinal imaging atlas reports desynchronized mitophagy changes during murine brain aging and highlights complex spatiotemporal dynamics in distinct subregions and cellular contexts.

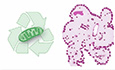

## Teaser

The selective turnover of mitochondria via autophagy, known as mitophagy, is a major quality control mechanism safeguarding eukaryotic cells, including highly specialized neurons in the mammalian brain. A new resource by Rappe et al (2024) now provides a spatiotemporal landscape of mitophagy changes during mouse brain aging at unprecedented resolution, encompassing different cell types and subregions, and revealing unanticipated, context-specific dynamics.

The “*mito*-QC” reporter is a transgenic mouse carrying a pH-sensitive fluorescent-tag fused to FIS1, a protein of the outer mitochondrial membrane. When introduced to the field eight years ago, it opened a window into the lysosomal turnover of mitochondria within different tissues and organs (McWilliams et al, 2016). It was readily appreciated that in the long-lived postmitotic neurons of the mammalian brain, as well as in supporting astrocytes and microglia (McWilliams et al, 2018), mitophagy occurs constitutively under physiological conditions and resting states, therefore representing a homeostatic process. This was an important milestone, given that mitophagy was primarily studied in mammalian cells under non-physiological conditions entailing oxidative stress and mitochondrial damage.

Neurons are mostly born during embryonic development, and as they are terminally differentiated cells, they need to survive the lifetime of the organism, which in the case of mammals can be a century. As such, it is not surprising that even under physiological states, they need to maintain tight control over the number and quality of their extensive mitochondrial networks to ensure sufficient energy supply and protect themselves against hazardous reactive oxygen species that originate from mitochondria. With mitochondrial dysfunction emerging as a key feature of age-related neurodegenerative diseases, mitophagy has been intuitively expected to decline with aging within brain cells. This expectation was also led by work in invertebrate models, convincingly demonstrating declining mitophagy with aging in the nervous system and other tissues of the nematode *C. elegans* and further implicating mitophagy in lifespan extension, at least in long-lived models (Palikaras et al, 2015). In addition, a neuron-specific and adult-onset overexpression of *parkin or Bnip3*, genes encoding for two well-characterized mitophagy receptors, was sufficient to extend the lifespan of another invertebrate model, *Drosophila melanogaster* by boosting mitophagy (Schmid et al, 2022). However, whether a similar notion holds true in the mammalian brain has remained a debated topic with contradicting results (Jimenez-Loygorri et al, 2024; Sun et al, 2015).

**Figure 1:**
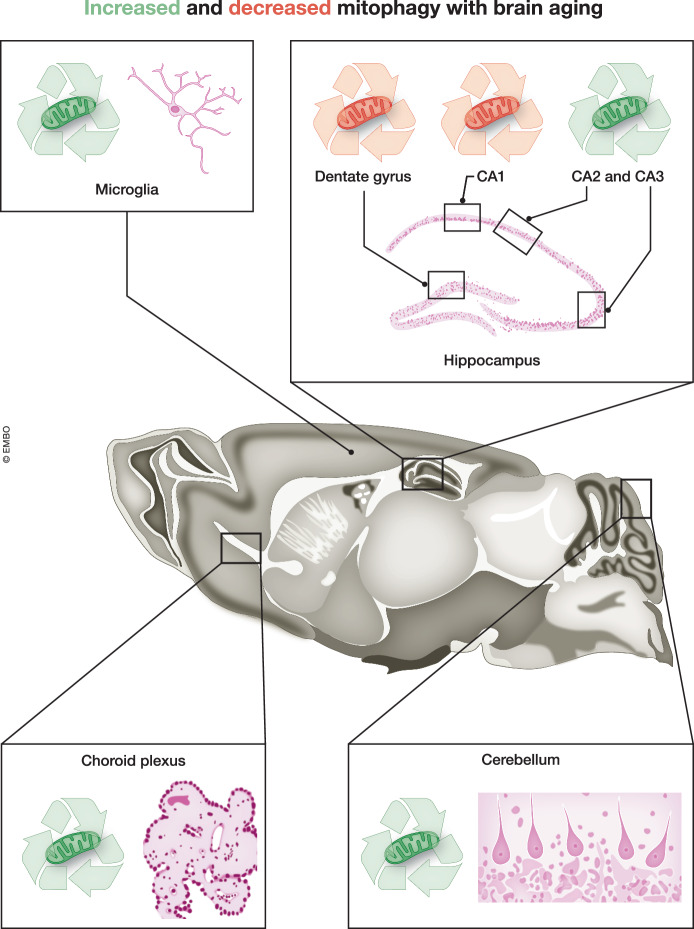
Spatiotemporal landscape of mitophagy changes during mouse brain aging. The use of mitoQC mice to study the density of mitolysosomes, allows for dynamic in vivo mapping of mitophagy in long-lived postmitotic neurons and glial cells of the aging mammalian brain. Desynchronised patterns of exemplary cell types and subregions with context-specific dynamics are highlighted.

The current work by Rappe et al (2024) has now used the mitoQC reporter mouse to study the density of mitolysosomes, namely mitochondria that have been delivered to lysosomes, in different types of brain cells and at different postnatal ages, ranging from young to geriatric mice. In parallel, they also assess the density of autolysosomes in the same cells and timepoints, using the autoQC reporter, a transgenic mouse where the autophagic marker LC3B is N-terminally tagged with a pH-sensitive fluorescent tag, allowing the monitoring of all LC3-positive autophagosomes irrespective of their cargoes. Their findings reveal a more complex regulation of mitophagy in the mammalian brain than the one anticipated based on invertebrate studies. Namely, age-related changes in mitophagy do not necessarily entail a decrease with aging and they occur in a cell-context dependent as opposed to a widespread manner (Fig. 1).

To give some examples, striking differences are observed within different regions of the hippocampal formation, which are physically very proximal to each other. Notably, while there’s a sharp age-related decline in mitophagy within neurons of the CA1 region and of the dentate gyrus, mitophagy appears largely stable in the CA2/3 regions, whilst an overall decline of autophagy, evidenced by the autoQC reporter, is observed across the hippocampus. Similarly opposing trends are seen for nigrostriatal and ventral tegmented area (VTA) dopaminergic neurons, the former showing increased mitophagy with aging and the latter a peak in middle age that declines in aged mice. By contrast, the cerebellum features as a brain region where all cell types examined, including Purkinje cells, astrocytes and microglia show continuously increasing mitophagy levels with aging. As a conclusion, there seems to be no general synchronization of mitophagy dynamics based on brain region, neither on neurotransmitter type, making it a landscape that is likely influenced by integrated parameters of cell identity and local state. On the other hand, the fact that microglial cells, the main immune resident cells of the brain, show increasing mitophagy with aging across different brain regions, invites the speculation that mitophagy in these cells may be implicated in an activated immune response in aging that requires further investigation.

From an evolutionary perspective, the absence of a generalized and widespread decrease in mitophagy in the mammalian brain may be related to some fundamental differences between mammals and the invertebrate genetic models previously examined. One main difference is that mammals are generally long-lived compared to invertebrates, meaning that they exhibit slower aging rates. This holds particularly true for terminally differentiated neurons which represent some of the longest-lived cells within the mammalian body and are therefore expected to age at slower rates compared to dividing cells. Another parameter may be differential metabolic rates and energetic requirements between the nervous systems of mammals and invertebrates, suggesting a greater dependence of the mammalian nervous system on mitochondrial plasticity and turnover. Finally, in invertebrates, the association of the nervous tissue with specialized microglia and other macrophages is not completely understood. In contrast, neuroimmune interactions feature strongly in the aging mammalian brain, and mounting evidence suggests their role in neuronal energetics (Chausse et al, 2024). Therefore, taken together, an increase in mitophagy may be beneficial for neurons to fence off inflammatory and other immune challenges with aging and to maintain their energetic needs for longer.

The cellular mechanisms underlying mitochondrial turnover and quality control are intensely investigated. There is mounting evidence that rather than degrading whole mitochondria as it is perceived in classical mitophagy, individual protein complexes or compartments of mitochondria can be selectively degraded in a process known as piecemeal mitophagy. This process is thought to entail the budding off of mitochondria-derived vesicles that are sequestered by the autophagic machinery, and has been implicated in the degradation of the SAM and MICOS complexes that reside on the outer and inner mitochondrial membrane, respectively (Abudu et al, 2021; Le Guerroue et al, 2017). Recent work conducted in mouse embryonic fibroblasts, demonstrated the piecemeal removal of patches of the inner mitochondrial membrane directly by lysosomes in a microautophagy-like manner and without any lysosomal uptake of mitochondrial matrix or outer membrane (Prashar et al, 2024). The presence of piecemeal mitophagy processes in the brain remain elusive, yet fascinating as a concept. It is unknown whether the mitoQC reporter mouse, which is designed by tagging an outer mitochondrial membrane protein, can also monitor these processes or excludes some forms that entail the exclusive degradation of inner mitochondrial membrane segments and complexes.

Thus, this state-of-the-art implementation of the mitoQC reporter mouse to study brain mitophagy across space and time, has paved the way towards a deeper understanding of the principles of mitochondrial turnover in the mammalian brain. It also forms a solid base for incorporating newly emerging mechanisms of mitochondrial turnover within brain physiology and aging.
